# Protection against Fire in London

**Published:** 1907-12-14

**Authors:** 


### PROTECTION AGAINST FIRE IN HOSPITALS.

We are informed by the hon. secretaries of King Edward's Hospital Fund for London that the Prince of Wales, as President of the Fund, having requested Sir Eyre Shaw and Captain Wells to draw up recommendations as to the best method of securing protection against fire in the various London hospitals, these gentlemen have made the following report:—

On February 11 last your Royal Highness was pleased to appoint us a committee for the purpose of drawing up a few general rules which might be circulated by the Committee of King Edward's Hospital Fund to the governing bodies of all the various hospitals, with a view to assisting them to secure a greater degree of protection against fire.

We have the honour respectfully to submit the following report, embodying the result of our deliberations under the foregoing reference.

In submitting suggestions for safety from fire which would be generally applicable it must be plainly stated that such suggestions to be practicable must be very few in number, as otherwise many of the most obvious and important of them would not be applicable to more than a few buildings.

There has never been a general or generally recognised plan of constructing hospitals; on the contrary, all that we have ever seen have been situated apparently where ground happened to be available, whether suitable or not, and to have been constructed by different architects or builders on totally different plans, or even in some cases by the same architects or builders but on different plans, although in the same town or city. For this want of system there can probably be no remedy, as each case must depend on local requirements and conditions—especially local finance.

For these reasons it is absolutely necessary to say that, although in any one case there may be no difficulty in making a complete and exhaustive report upon which recommendations might be based, such a report could not be made applicable to others, as in each case the individual circumstances would have to be specially and separately considered.

Here we may, however, remark incidentally that in future construction it would be an advantage if the site and architectural considerations could, to some extent at any rate, be influenced by the Committee of the Fund, with special reference to safety of life in case of fire or panic; and to ensure for future hospitals some "system" in building.

Cleanliness and freedom from dust are, of course, absolutely essential for reducing risks of fire; and in one important point hospitals have an advantage over almost all other public institutions in having virtually a watch on every floor in the shape of nurses who are never absent.

We repeat that exhaustive recommendations in the case of any individual building could only be drawn up upon a report, such as would be made by an experienced fireman, upon the particular institution. But we enclose in an appendix a series of suggestions framed entirely with the view of helping existing authorities in existing circumstances, while we do not entertain the idea of responsibility either for the Committee of the Fund or for ourselves. It must again be clearly stated that in many of the smaller or older hospitals no steps in the way of safety are possible except with great care and cleanliness on the part of officials, unfailing watchfulness for the first symptom of danger, however slight, and immediate notice to all concerned, both inside and outside the premises.

### APPENDIX.

The Appendix is divided into two parts. The first, A, consists of points which should be borne in mind by hospital committees when framing regulations:—

1. It is important to have some arrangement for summoning those who should be alarmed. In arranging the method for alarm, it is necessary to avoid startling the patients unnecessarily. Police whistles distributed are worth consideration, and would have the effect of summoning police aid as well. Every endeavour should be made, however, only to disturb the affected area.

2. Each building containing patients should have means of immediately despatching information to the nearest fire brigade or police-station, and the nurses or others in charge should be instructed as to the proper mode of instantly sending for this external aid without waiting to see whether their own local appliances are sufficient or not. If the hospital is not on the telephone, the staff should know where the nearest fire-alarm post in the street is located and how to use it. This information should be given on the card of printed regulations. In order to save expense, an offer might be made to the fire brigade authority to allow a public fire-alarm to be placed at the hospital gate, which would be available for use by the public as well as by the hospital officials. If the hospital is on the telephone, the London Fire Brigade should be called by telephone and by fire-alarm post in the street. The following slip should be posted near the telephone, if any :—"In Case of Fire.—Ring up in the usual way. Say, 'Put me on to the fire brigade.' When answered, say, 'Fire at —— Hospital, —— Street,' etc., and any detail that your presence of mind dictates, namely large, small, roof, basement, etc."

3. The regulations of the hospital should provide organisation that will immediately stay "panic" and guide the patients to safety. If alternative staircases are not available, anything like a *cul de sac*, where several are accommodated, particularly on upper floors, requires special attention and consideration.

4. Every hospital should have within its walls some "first-aid" apparatus to deal with the outbreak. As the probable position of any outbreak can generally be foretold, the risks should be located and studied beforehand. Roof, kitchen, storerooms, cupboards, heating-places, workshops, laundry, etc., are examples of likely danger points. Fire-places and guards, gas-fittings (especially swinging gas-fittings), curtains, rubbish-collections, and places where candles and matches are used near clothing and bedding should also be studied. Fire drills adapted to the special dangers of the particular hospital should be quietly arranged periodically. This location of risks is the essence of effective fire drill. Certain of the staff should be detailed to handle the hospital fire appliances, and they should know this is their job. They should be so selected that some will always be present. The whole number should periodically be summoned to the different "fire risks" for drill. An ordinary mop and bucket will prove useful, in the early stages, to splash water, and any one can use them. Should the fire be due to electricity or upset of oil, be careful in applying water, but rather endeavour to smother with sand or wet blanket till the brigade arrives.

5. Printed instructions should be posted up freely, so that all are reminded that fire is a possible contingency. These instructions should deal with all the foregoing points, with any additions or amplifications appropriate to the particular hospital or to its appliances.

The second part, B, contains the following points, which should be remembered by the staff when an alarm takes place:—

1. On the discovery of a fire or on smelling smoke the staff and employees should not run about aimlessly or shout or shriek, etc., but quietly think and act on the instructions for raising the alarm.

2. The first object is to prevent "panic" among the patients and visitors, if any.

3. If the fire brigade has been called, the actual fire damage should not be much in the thoughts of the staff. It is imperative to see to the removal of patients who are in danger to safety, which is, as a rule, out of the smoke area, and preferably outside the building involved if a comparatively small one, or into a separate block if a large one. In a densely smoky atmosphere a wet flannel over the face is beneficial.

4. Those detailed for appliances should go to their duty at once, and endeavour to check the progress of the fire. It would be as well also for some one in authority on the spot to detail those who are to remove patients, and also those who are to receive them and see to their proper clothing and comfort.

N.B.—As far as the details of the actual steps to be taken when an alarm takes places are concerned, the printed regulations drawn up by the individual hospital, and the periodic fire drills, should ensure that every member of the staff knows what is his or her duty in the event of an alarm of fire.

